# Combined novice, near-peer, e-mentoring palliative medicine program: A mixed method study in Singapore

**DOI:** 10.1371/journal.pone.0234322

**Published:** 2020-06-05

**Authors:** Lalit Krishna, Kuang Teck Tay, Hong Wei Yap, Zachary Yong Keat Koh, Yong Xiang Ng, Yun Ting Ong, Sushma Shivananda, Scott Compton, Stephen Mason, Ravindran Kanesvaran, Ying Pin Toh

**Affiliations:** 1 Academic Palliative & End of Life Care Centre, Palliative Care Institute Liverpool, University of Liverpool, Liverpool, United Kingdom; 2 Cancer Research Centre, University of Liverpool, Liverpool, United Kingdom; 3 Division of Cancer Education, National Cancer Centre Singapore, Singapore, Singapore; 4 Division of Supportive and Palliative Care, National Cancer Centre Singapore, Singapore, Singapore; 5 Yong Loo Lin School of Medicine, National University of Singapore, Singapore, Singapore; 6 Duke-NUS Medical School, Singapore, Singapore; 7 Centre of Biomedical Ethics, National University of Singapore, Singapore, Singapore; 8 Lee Kong Chian School of Medicine, Nanyang Technological University, Singapore, Singapore; 9 Division of Medical Oncology, National Cancer Centre Singapore, Singapore, Singapore; 10 Department of Family Medicine, National University Health System, Singapore, Singapore; Nazareth Hospital, UNITED STATES

## Abstract

**Introduction:**

An acute shortage of senior mentors saw the Palliative Medicine Initiative (PMI) combine its novice mentoring program with electronic and peer mentoring to overcome insufficient mentoring support of medical students and junior doctors by senior clinicians. A three-phased evaluation was carried out to evaluate mentees’ experiences within the new CNEP mentoring program.

**Methods:**

Phase 1 saw use of a Delphi process to create a content-valid questionnaire from data drawn from 9 systematic reviews of key aspects of novice mentoring. In Phase 2 Cognitive Interviews were used to evaluate the tool. The tool was then piloted amongst mentees in the CNEP program. Phase 3 compared mentee’s experiences in the CNEP program with those from the PMI’s novice mentoring program.

**Results:**

Thematic analysis of open-ended responses revealed three themes–the CNEP mentoring process, its benefits and challenges that expound on the descriptive statistical analysis of specific close-ended and Likert scale responses of the survey. The results show mentee experiences in the PMI’s novice mentoring program and the CNEP program to be similar and that the addition of near peer and e-mentoring processes enhance communications and support of mentees.

**Conclusion:**

CNEP mentoring is an evolved form of novice mentoring built on a consistent mentoring approach supported by an effective host organization. The host organization marshals assessment, support and oversight of the program and allows flexibility within the approach to meet the particular needs of mentees, mentors and senior mentors. Whilst near-peer mentors and e-mentoring can make up for the lack of senior mentor availability, their effectiveness hinges upon a common mentoring approach.

To better support the CNEP program deeper understanding of the mentoring dynamics, policing and mentor and mentee training processes are required. The CNEP mentoring tool too needs to be validated.

## Introduction

Novice mentoring dominates mentoring in Palliative Medicine (PM) enhancing the personal, academic and professional development, clinical and professional practices and research productivity of mentees and mentors and boosting the reputations of host organizations [1–22]. Defined as a *dynamic*, *context-dependent*, *goal-sensitive*, *mutually beneficial relationship between an experienced clinician and a junior clinician (or medical student)*, *with the intention of supporting the development of the mentee*, novice mentoring’s success is built upon the provision of timely, specific, appropriate, holistic, accessible and longitudinal support to nurture personalised mentoring relationships [21]. However, limited resources, variations in mentoring approaches and a shortage of trained and experienced mentors have jeopardized mentoring processes [23–40] and raised the possibility of ethical concerns in mentoring [41–64].

To circumnavigate some of these concerns, a combination of near-peer or peer and e-mentoring to supplement the prevailing novice mentoring approach is proposed. Lim *et al*. [65] define near-peer or peer mentoring (NP mentoring) as a “*voluntary collaboration between colleagues of similar rank and experience and common academic interests on mutually beneficial structured fixed term projects*. *These processes often include a senior clinician who facilitates discussions*, *provides personalized support and feedback and oversees the mentoring process*. *Effective NP mentoring nurtures long-term friendships and professional collaborations between peers*”. Chong *et al*. [66] define e-mentoring as a “*personalised*, *internet or electronically mediated approach that is largely used to complement face-to-face mentoring to provide personalised*, *appropriate*, *specific*, *timely*, *holistic*, *accessible and longitudinal mentoring support to build mutually beneficial mentoring relationships between the host organization*, *a senior mentor and an individual mentee*. *Working within the confines of prevailing professional codes of conduct and standards of practice this approach is focused upon realizing the goals and needs of the mentee*, *the mentor*, *the host organization that supports and oversees the program and their relationships*. *Its asynchronous nature also nurtures reflective practices that helps develop deeper mentoring relationships*”. Whilst Chong *et al*. [66]’s systematic review of e-mentoring, Tan *et al*. [5]’s systematic review of peer and near peer mentoring and Lim *et al*. [65]’s proposal for the use of a combination of novice mentoring and peer and near-peer mentoring, suggest that supplementing novice mentoring with near peer and e-mentoring will provide better oversight and timely, appropriate, personalised, specific, holistic and longitudinal support of mentoring relationships and mentees, combining novice, near peer/peer and e-mentoring to create Combined Novice, E-mentoring and Peer mentoring or CNEP mentoring remains untested [3, 32, 38–40, 67, 68].

However in the face of a sudden shortage of mentors following the untimely demise of a senior mentor and the sabbatical by another senior mentor, the Palliative Medicine Initiative (PMI), a research-based novice mentoring program hosted by the Division of Supportive and Palliative Care (DSPC) at the National Cancer Centre Singapore (NCCS), had to put theory to the test [21]. Building on data from Chong *et al*. [66]’s, Tan *et al*. [5]’s and Lim *et al*. [65]’s reviews the PMI established a CNEP program to maintain mentoring support for the 28 medical students affected by this sudden loss of mentors.

### The PMI’s CNEP program

The PMI program was designed to increase student-led research in palliative medicine, professionalism, medical ethics, medical education, end-of-life ethics (EoLE) and health services through use of a novice mentoring approach. To do so, the PMI provided mentoring support to medical students and junior doctors through the various stages of the research and publication process. This homegrown approach was designed on the team’s own evaluation of prevailing data which later formed the basis for systematic reviews and scoping systematic reviews around the key elements of mentoring relationships [3], mentoring structure [6] and the mentoring environment [19].

By inculcating new novice [4, 7, 9] mentoring data, the PMI has evolved over time. However, at its centre is a flexible structure, approach and duration dependent on the specific research objectives, mentee’s abilities, motivations, goals and availabilities, the mentor’s goals, ability to support the mentee, experience and training, the progress of the research and mentoring project and the publication process. This process often ran between 6 months to 3 years in duration depending on the time taken to complete the publication of the research project in a peer reviewed journal. In Krishna *et al*. [21]’s account of the PMI program, there was a significant number of mentoring relationships that continued long after the completion of the primary research project with many mentees taking up new research projects under the PMI program.

This mix of flexibility and consistency within the mentoring approach has seen the PMI program publish over 50 mentored articles in peer reviewed journals and the presentation of more than 70 mentored posters in international medical education, medical ethics and Palliative Medicine conferences over the last 9 years [21].

The new CNEP mentoring approach supplements the PMI’s prevailing novice mentoring program with trained near peer-mentors who had successfully completed the PMI program. CNEP mentees and near peer mentors were also provided with access to e-mentoring options such as email, WhatsApp^®^ (Facebook, Inc., Menlo Park, California, United States of America), text messaging, Facetime^®^ (Apple Inc., Cupertino, California, United States of America) and Skype^®^ (Microsoft Corporation, Redmond, Washington, United States of America) meetings with the senior mentor to ensure timely, specific, appropriate, personalised, longitudinal, accessible and holistic support and feedback. A brief description of the new CNEP program is enclosed in Table 1.

**Table 1:** The PMI CNEP mentoring approach.

1. Near peer mentors within the CNEP mentoring program work with senior mentors to provide mentees with research advice, practical guidance and moral support. These interactions maybe carried out face-to-face or via e-mentoring platforms.
2. The near peer mentors involved in the CNEP program are mentees who exhibited good mentoring and research skills, have published numerous articles as first author and/or corresponding author, and participated in oral and poster presentations at various local and international conferences as part of the PMI program.
3. All the senior mentors recruited to the PMI were experienced clinicians and of consultant or attending grade at the DSPC and were registered Palliative Medicine specialists with the Singapore Medical Council.
4. All senior and near-peer mentors were provided with mentor training to ensure a consistent CNEP mentoring approach [69–71].
5. Mentees were briefed on the basic codes of conduct set out by the program, the mentoring approach adopted and the roles and responsibilities of the mentee, the mentor and the near peer mentor.
6. Mentees were also briefed on the professional, mentoring and research interests of the 2 female and 2 male PMI senior mentors and provided guidance on how to select a mentor. The PMI offered all medical students the opportunity to initiate one-to-one mentoring relationships with one of 4 PMI senior mentors at DPM during their 2-week Palliative Medicine electives. Mentee-initiated matching was promoted to create enduring and personalized relationships [72–74].
7. The near-peer mentors were allocated to the respective mentoring projects and teams by the senior mentors.
8. Once the mentees, mentors and near peer mentors decide to work together they are invited to pre-mentoring meetings to align expectations and agree upon a code of conduct, roles and responsibilities and the timelines for the project and determine the frequency and types of meetings that will be employed over the course of their particular mentoring relationship [75–77].
9. Near-peer mentors are trained by the senior mentor on the mentoring approach, workflow, the standards expected of them and the code of conduct.
10. The senior mentor closely monitored the mentoring relationship through separate monthly meetings with mentees and near-peer mentors. These meetings were usually face-to-face and in person and are carried out in private to allow the mentees and near-peer mentors the chance to discuss their concerns in a safe environment.
11. Both mentees and near peer mentors can also access the senior mentors at any time using the e-mentoring platform or arrange face-to-face meetings to discuss any potential conflicts, poor progress in the mentoring process and breaches in the codes of conduct.
12. Unlike most programs the PMI’s CNEP program does not provide mentees and near-peer mentors with ‘protected time’ and often require meetings to occur in the mentee’s and near-peer mentors’ spare time with only two weeks of their involvement in the program given academic recognition by the university.
13. Senior mentors are provided with ‘protected time’ to pursue their education and mentoring projects [78].
14. Contributions to the PMI program and mentoring successes were considered in the senior mentor’s yearly appraisals and in applications for promotion and academic credentialing [71, 73, 79–81]. PMI mentors are also given priority for funding and leave for education meetings and conferences.

**Fig 1:**
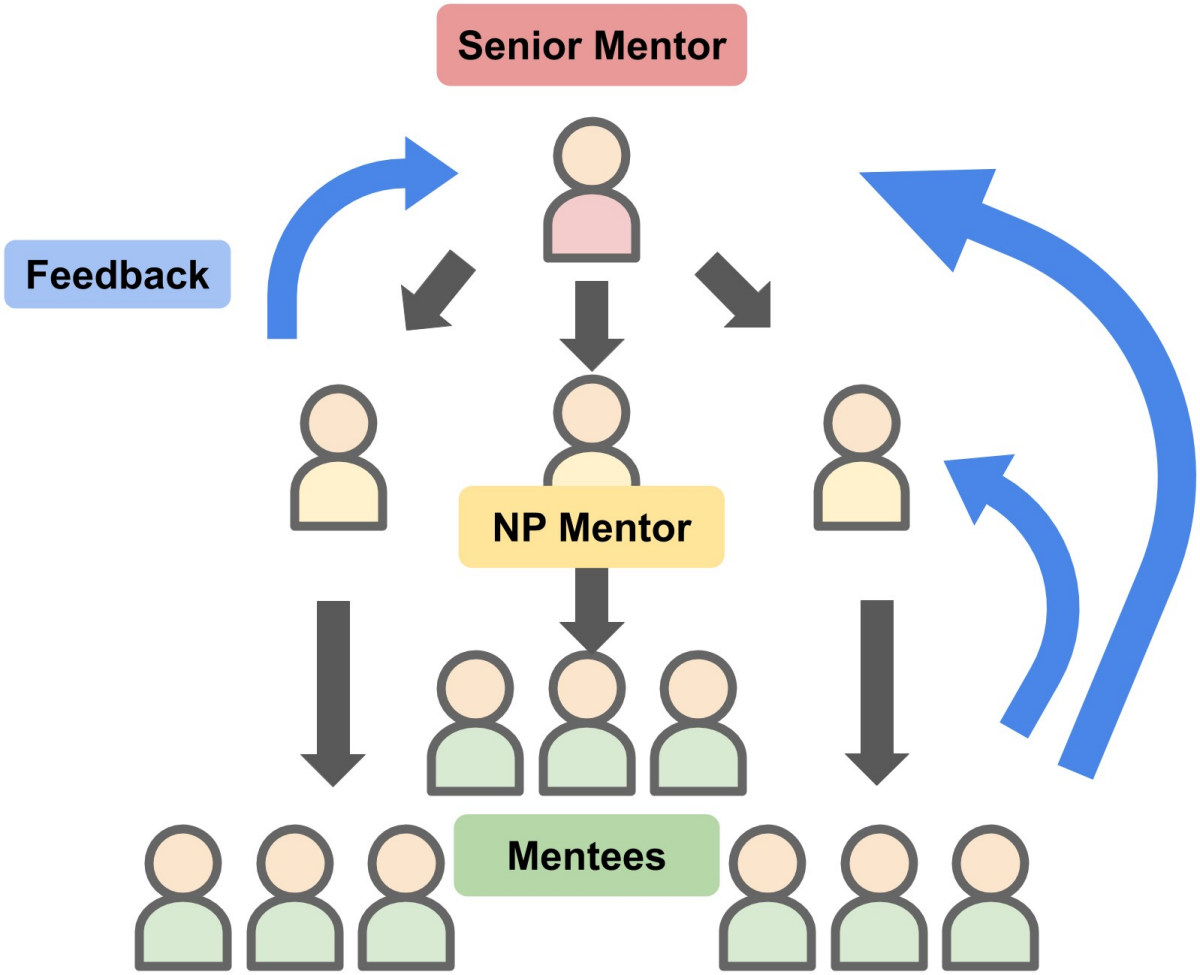
Organisation structure in PMI.

The organisation structure of senior mentors, near-peer mentors and mentees may be found in Fig 1 below. Each senior mentor advised a group of near-peer mentors, who then in turn advised a group of mentees. Mentees and near-peer mentors may give feedback privately to the senior mentor, and mentees may also give feedback privately to their near-peer mentors.

### Evaluating the CNEP program

Given the unproven benefits of CNEP mentoring, an evaluation of CNEP mentoring was called for. The research team consisting of a methodologist, an educationalist, a senior librarian, 2 senior clinicians, 4 freshly recruited medical students with no research experience and 4 junior doctors and 4 medical students with previous research experience in the PMI novice mentoring program discussed the issues pertaining to assessing CNEP mentoring with educationalists, clinicians, academics, mentors and administrators at Duke-NUS Medical School, Yong Loo Lin School of Medicine at the National University of Singapore, the University of Liverpool, the National Cancer Centre Singapore and Singapore General Hospital (henceforth the expert team) to delineate the research questions. The primary research question was “what were the mentee’s experiences of the CNEP mentoring program?” and the secondary question was “what was the impact of the addition of e-mentoring and near peer mentoring to the PMI novice mentoring program?”.

However, there were significant limitations in addressing these research questions in the absence of effective assessment tools to evaluate mentee experiences in CNEP mentoring. To overcome this issue the expert and research teams concluded that a new tool to assess mentoring was required.

## Methods

**Fig 2:**
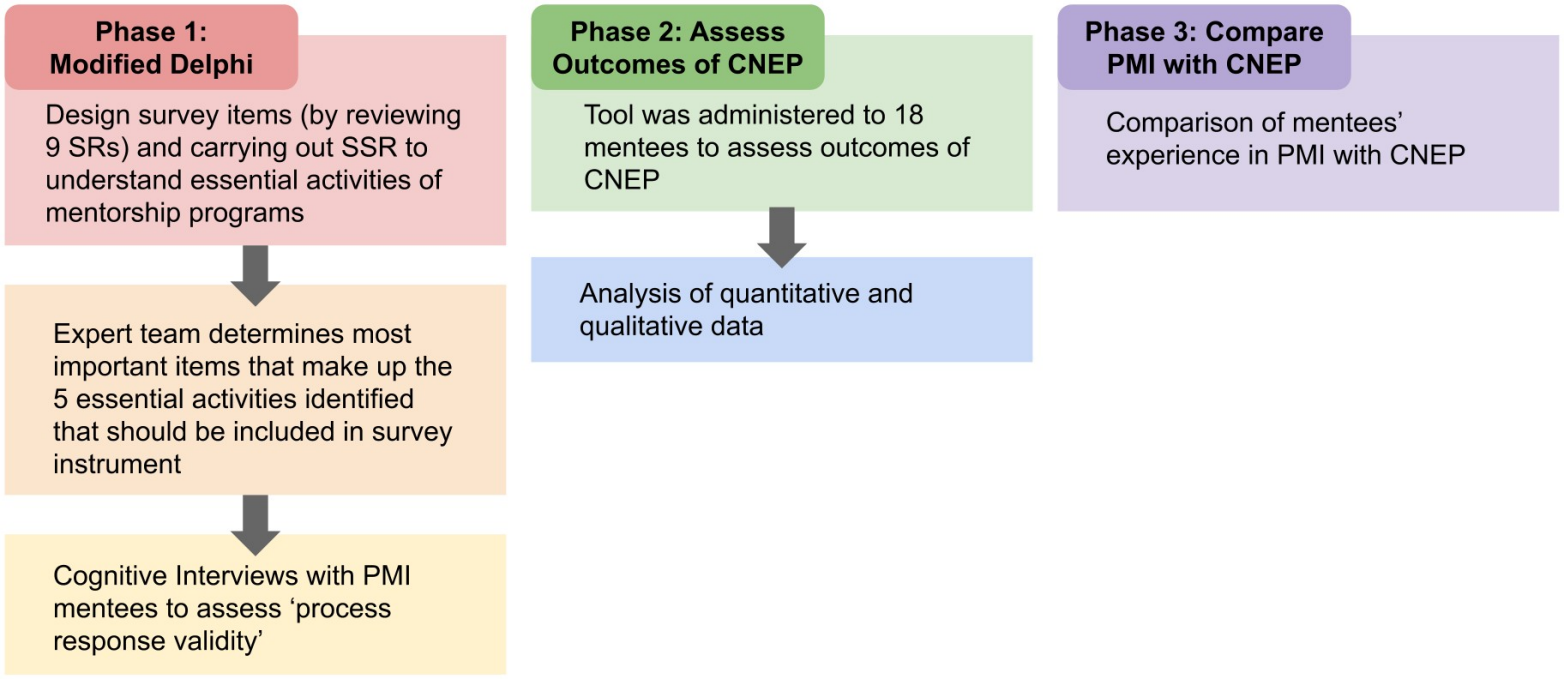
Methodology of this study.

A three phased approach was proposed to design a new tool and to address the primary and secondary research questions. Phase 1 involved design of a new tool, Phase 2 involved assessing the mentee’s experiences using the newly designed CNEP mentoring tool and Phase 3 involved comparing the findings of the new tool with data from the PMI novice mentoring program. This process is outlined in Fig 2 below.

### Phase 1

A Delphi study [82] was proposed to design a content-valid instrument to obtain mentees’ perceptions of their mentoring experiences and outcomes in the CNEP program.

The first step of the Delphi study sought to provide a set of *survey* questions that would be used to form the items of the Delphi survey questionnaire [82]. To do so the research team reviewed nine systematic reviews on novice, near-peer, peer and e-mentoring programs [1, 3–6, 12, 13, 19, 20]. In addition, the research team also carried out a systematic scoping review of mentoring tools to extract specific activities identified as being essential in mentorship programs (this will be discussed elsewhere). The findings of the review of these reviews revealed that 5 essential activities in mentoring programs which were 1) matching practices, 2) mentoring relationships, 3) the mentoring environment, 4) the mentoring structure and 5) the mentoring culture.

These 5 essential activities were characterised by a total of 168 activities that were reviewed for redundancies, trimming the list to 67 items [83]. This list of 67 items was then submitted to a purposive sample of 9 mentorship “experts” who have published on mentoring in medical education or who are acknowledged experts in medical education. These experts who were not part of the expert team then rated each of the 67 items for their importance to include in a survey related to measuring the five domains. Experts were asked to rate the item in one of the following four ways: “do not include, present to mentors only, present to mentee only or present to both mentor and mentee”.

A 70% consensus agreement for each item’s inclusion was set a-priori by the research team. Items with more than 70% of participants agreeing that a question should be addressed to either the mentor only, mentee only, or mentor and mentee were eligible for inclusion into the final survey instrument. After the first round of ratings, the number of items was reduced to 33. The items were then formatted into a final survey instrument and sent back to the experts for their assessment of how well each set of items reflected the domain of interest. The final survey received unanimous support from all 7 experts who participated in the second round.

The second stage in the Delphi study involved cognitive interviews with 3 PMI mentees. Cognitive Interviews served to assess the ‘response process validity’, or how prospective participants interpret items within the pilot tool [84] and whether the participant’s interpretation of the questions matches what the survey designer had in mind [85, 86]. These Cognitive Interviews were carried out using a think-aloud approach that was audiotaped and reviewed by a trained interviewer who was also an independent clinician with whom the participants have not worked with before. The ‘think-aloud’ technique required participants to explicitly ‘think aloud’ as they answered the questionnaire to help elucidate how a response was arrived at. The ‘think-aloud’ technique was supplemented with ‘verbal probing’, where questions were asked to ‘probe’ or delve deeper into the basis for a response. This process allowed for open-ended and unanticipated information to be gathered. Minor modification in the wording of the survey instrument were made following the first round of the Cognitive Interviews. In the second round of Cognitive Interviews 3 other mentees were asked to adopt the ‘think-aloud’ technique as they completed the modified survey. Analysis of their experiences revealed no new issues compared to those highlighted in the first round. As a result no substantive changes were made to the survey instrument following the second round.

### Phase 2

In Phase 2 the new tool was piloted amongst mentees in the CNEP program. Email invites containing information about the study, the tool and an attached informed consent form was sent to 28 mentees who had completed the CNEP program and had published articles in peer reviewed journals and or had presented posters in conferences between 2016 and 2018. Invitees who agreed to participate in the survey printed out, signed, and scanned or photographed the consent form and returned it to a study team member with whom they have not worked with previously and who is not part of the team analysing the data. Upon receipt of the signed consent form, the study team member then sent a link to the survey that was hosted on a university website.

This study was approved by the Central Institutional Review Board of the National Cancer Centre Singapore *(CIRB Ref* *2018/2904*: *Creating a new tool to evaluate Combined novice*, *peer*, *near-peer and e-mentoring (C-NEP) mentoring*).

#### Data collection and analysis

Overall, 18 of 28 of those invited to participate completed the study (10 male and 8 female medical students and postgraduate medical doctors, who began their participations in the PMI at varying years of study in the 5-year undergraduate medical degree at National University of Singapore and Nanyang Technological University). The profile of mentees who participated in the study can be found in Table 2 found in the results section.

**Table 2:** Mentee’s profile.

**Age/ years old**	**n (total = 18)**	**n/ %**
20	2	11%
21	2	11%
22	2	11%
23	4	22%
24	1	6%
25	3	17%
26	3	17%
27	1	6%
**Gender**	**n (total = 18)**	**n/ %**
Male	10	56%
Female	8	44%
**Years since graduating from medical school**	**n (total = 18)**	**n/ %**
Not graduated yet	9	50%
Post-Graduate Year 1	5	28%
Post-Graduate Year 2–4	4	22%
**Purpose(s) of Mentoring**	**n**	**n/ %**
Research	17	94%
Clinical Training	5	28%
Academic Advancement	6	33%
Psycho-emotional Support	8	44%
Career Progression	10	56%
**Setting(s) in which mentoring takes place**	**n**	**n/ %**
Hospital	11	61%
University	16	89%
Community Health Care Settings	2	11%

#### Analysis of the qualitative open-ended survey responses

Three reviewers (YPT, KTT, LK), experienced in the use of Braun and Clarke [87]’s approach to thematic analysis carried out independent analysis of the anonymized qualitative data collected from the open-ended questions. To enhance rigour, analysis of the qualitative data focused upon descriptions of PMI experiences to preserve the participant’s ‘voice’ and the integrity of their ideas, emotions, and beliefs. An inductive approach allowed themes to be “inductively defined from the raw data without any predetermined classification” [88].

In keeping with the first phase of Braun and Clarke [87]’s approach, an iterative step-by-step thematic analysis was carried out. By immersing themselves in the data and making notes, the reviewers sought to find meaning and purpose in the data.

Next, the reviewers constructed ‘codes’ or a “feature of the data (semantic content or latent) that appears interesting to the analyst, and refer to ‘the most basic segment, or element, of the raw data or information that can be assessed in a meaningful way regarding the phenomenon’ [89] from the ‘surface’ meaning of the mentee’s responses. The initial codes from ‘open coding’ were then grouped into categories according to their similarities.

In the third phase of Braun and Clarke [87]’s approach categories were organised into themes that best depict the data. Three reviewers (YPT, KTT, and LK) used mind maps to illustrate the links between the various codes and to help delineate themes whilst the other reviewer employed lists (RK) to identify potential themes which “represents some level of patterned response or meaning within the data set”.

In the fourth phase of Braun and Clarke [87]’s approach each reviewer reviewed and refined their themes to ensure they were coherent and representative of the whole data set.

In this fifth phase of Braun and Clarke [87]’s approach, the reviewers continued to work independently naming and delineating the specific characteristics of each theme. The three reviewers used Sambunjak *et al*. [90]’s “negotiated consensual validation” approach to agree upon a common coding framework and code book. The code book consisted of the codes, sub-themes, definitions, descriptions of terms and guidelines on when to use and when not to apply [91].

The reviewers independently extracted and classified all quotations using the code books, collapsed them into themes and subthemes [92] and maintained an iterative approach to the analysis [93].

An external reviewer analysed each code for consistency and accuracy [92]. The themes identified by each reviewer were discussed online and at an author’s meeting where consensus on a final list of themes and subthemes was achieved using the “negotiated consensual validation” approach.

Quantitative analysis was limited given the small numbers of respondents.

#### Triangulation of qualitative and quantitative survey data

Descriptive analyses were performed on the quantitative survey data, reporting the proportion of Likert scale responses for the respective survey questions. The reviewers compared the quantitative results with the qualitative survey data to propose possible explanations for the quantitative survey responses, and highlight any contradicting data points between the qualitative and quantitative datasets [94].

#### Validity and reliability of the analysis

For the purposes of triangulation of coding and thematic analysis of qualitative data, the analysis was carried out by three independent reviewers. The codes and themes identified by each reviewer was discussed by the three reviewers in online and face-to-face meetings. In addition, the findings of each reviewer and their combined analysis was reviewed by an experienced external reviewer well versed in the topic at hand. To further ensure theoretical validation, the results of the analysis was compared with prevailing data. An iterative process was employed which meant that any new codes identified meant that all the survey data were reviewed to verify the classification and ensure complete data extraction.

#### Writing of the manuscript

This manuscript adheres to the SQUIRE guidelines [95].

## Results

### Demographic data

Thematic analysis of the responses to the open-ended questions in the survey revealed 3 themes including CNEP mentoring process, the benefits and the challenges of CNEP mentoring. The themes expand upon the descriptive statistical analysis of specific close-ended and Likert scale responses of the survey, which will be discussed in tandem.

### A. CNEP mentoring process

There are three sub-themes delineated–“mentoring stages”, “e-mentoring’s role” and the “near-peer mentoring’s role”.

#### 1) Mentoring stages

There are 6 subthemes to mentoring stages. These include recruitment, aligning expectations, mentoring process, mentoring relationships, mentoring environment and near peer role.

*i*. *Recruitment*. Eighty-nine percent (89%, n = 16) of mentees selected their own senior mentor whilst only 33% (n = 6) of mentees selected their near-peer mentor. Mentor selection was based upon a variety of factors. Prior interactions with the mentor and recommendations from colleagues were important considerations as were the mentor’s personal attributes, experience and common personal and professional interests (Table 3).

**Table 3:** Factors influencing participation in PMI and mentor selection.

		Not important at all		Moderately Important		Very Important	
		n	n/%	n	n/%	n	n/%
**How important are the following factors in influencing your decision to join the mentoring program?**	Mentor's Experience and Achievements	1	6%	9	50%	8	44%
	Prior Interactions with mentor	1	6%	4	22%	13	72%
	Standing and reputation of the mentoring program	2	11%	14	78%	2	11%
	Recommendations by colleagues	1	6%	8	44%	9	50%
**How important are the following attributes when choosing your mentor(s)?**	Mentor's professional interests	0	0%	11	61%	7	39%
	Mentor's personal interests	6	33%	10	56%	2	11%
	Skills & qualification	0	0%	12	67%	6	33%
	Experience	0	0%	6	35%	11	65%
	Personal attributes of mentor	0	0%	2	11%	16	89%

In most cases the near-peer mentor was often determined by the senior mentor (n = 12). The role of the senior mentor was determined by research, clinical and educational experiences (n = 18, Table 4).

**Table 4:** Perceptions of research and mentoring experiences at the start of the program.

**How much do you and your mentor differ in term of**:	**Significant difference**		**Some difference**		**No difference**	
	**n**	**n/%**	**n**	**n/%**	**n**	**n/%**
Research Experience of Senior Mentor	18	100%	0	0%	0	0%
Clinical experience of Senior Mentor	18	100%	0	0%	0	0%
Educational knowledge of Senior Mentor	18	100%	0	0%	0	0%
Mentoring skills and experience of Senior Mentor	18	100%	0	0%	0	0%
Research Experience of Near-peer Mentor	17	94%	1	6%	0	0%
Clinical experience of Near-peer Mentor	17	94%	1	6%	0	0%
Educational knowledge of Near-peer Mentor	17	94%	1	6%	0	0%
Mentoring skills and experience of Near-peer Mentor	16	89%	2	11%	0	0%

	**Have you (n)**			**Has your senior mentor (n)**			**Has your near-peer mentor (n)**		
	Yes	No	Unsure	Yes	No	Unsure	Yes	No	Unsure
Published any research?	1	17	0	17	0	1	18	0	0
Presented any research work formally?	3	15	0	17	0	1	17	0	1
Attended formal research skills training?	2	16	0	14	0	4	15	0	3
Been in a mentoring program previously?	0	18	0	14	0	4	16	0	2
Mentored others before?	2	16	0	17	0	1	17	0	1

In 89% (n = 16) of case mentees saw the senior mentor as the consultant/attending. The near-peer mentor was in most cases a senior resident or resident (n = 12, Table 5).

**Table 5:** Mentor’s stage of career.

		n	n/%
**Career Stage of Senior Mentor**	Consultant and above	16	89%
	Senior Resident/Resident Physician/Staff Registrar	2	11%
**Career Stage of NP Mentor**	Consultant and above	4	24%
	Senior Resident/Resident Physician/Staff Registrar	6	35%
	Junior Resident/Medical Officer	6	35%
	Medical Student	1	6%

*ii*. *Aligning expectations (n = 11)*. Once mentees and mentors agreed to work together, they met to align expectations (n = 18), availability (n = 18), goals (n = 15) and timelines (n = 12) and establish their roles and responsibility (n = 11, Table 6). Mentees saw this process as critical (n = 3) and as a means to “*clarify whatever doubt and expectations about the process beforehand*” (Mentee 17).

**Table 6:** Recruitment and matching process.

		Yes		No	
		n	n/%	n	n/%
**At the start of mentoring, I was asked about the following**	Abilities and achievements	7	39%	11	61%
	My expectations of the mentoring process	18	100%	0	0%
	My availability	18	100%	0	0%
	My mentoring goals	15	83%	3	17%
**At the start of mentoring, I was provided with the following**	Structured briefing on the mentoring process	13	72%	5	28%
	Clearly stipulated expectations, code of conduct, roles and responsibilities	11	61%	7	39%
	Clear timelines and action plans of the project	12	67%	6	33%
	Training for to prepare for mentoring	5	28%	13	72%
	Face-to-face meetings with mentor	17	94%	1	6%

*iii*. *Mentoring process*. The PMI CNEP mentoring program is built around a structured research process. This research-based approach guided the mentoring approach and ensured that it is easily understood by mentees, near-peer mentors and senior mentors as well as the host organization and applied in a consistent fashion. The regular steps involved in the research process including the design, data gathering, review of the study findings, analysis of the data, manuscript preparation, and ending with reflection of the research process ensured a systematic mentoring trajectory replete with clear stages that can be evaluated longitudinally.

*iv*. *Evolving mentoring relationship (n = 17)*. A key feature in the mentoring process was change. This was primarily the result of changing schedules and priorities, access to training and support and the inevitable difference in the focus of each stage of the research based mentoring process. The personal and professional needs of the mentee also changed the mentoring relationship (Table 7).

**Table 7:** Reasons that promote changes in mentoring relationship.

Our mentoring relationship has changed over time due to:	Yes		No	
	n	n/%	n	n/%
Changing needs	13	72%	5	28%
Change in schedule/availability	14	78%	4	22%
Change in access to mentor/mentee	11	61%	7	39%
Change in stage of development of mentee	17	94%	1	6%
Problems within the relationship	3	17%	15	83%
Ethical lapses	1	6%	17	94%
Change in priorities	6	33%	12	67%

As a result, there were regular reviews of milestones and discussions to resolve issues, realign goals and expectations, and provide specific trainings and resources to empower mentees in the research process.

*a) Review milestones* (n = 2)
  “It (mentee role) did (change), based on changes and needs of the relationship.”
  (Mentee 1)
*b) Resolve issues* (n = 3)
  “I am thankful that they are always there to patiently communicate ideas with me, remain open and honest in all interactions, and willing to clarify any misunderstandings so that my relationships with them continue to be built on trust.”
  (Mentee 15)
*c) Realign goals and expectations* (n = 1)
  “Set good deadlines and negotiate where needed.”
  (Mentee 4)
*d) Provide specific training and resources* (n = 1)
  “to be proactive and communicate well with mentors on goals, responsibilities and expectations”
  (Mentee 18)

*v*. *Mentoring environment (n = 17)*. Change within the mentoring relationship however was possible given the impact of the mentoring environment and the mentee’s own attitudes and motivations (Table 8).

**Table 8:** Attitudes towards mentoring experience.

Throughout my mentoring experience,	Yes		No	
	n	n/%	n	n/%
I felt a sense of belonging, collegiality, and teamwork.	17	94%	1	6%
I was actively engaged in the mentoring process.	15	83%	3	17%
I was self-directed in learning.	13	72%	5	28%
I felt safe and supported.	16	89%	2	11%

The mentoring environment also helped support the mentees during trying times particularly in the face of competing commitments (n = 16), psychological and emotional issues (n = 9) and in addressing their professional identities along the course of the mentoring process (n = 9, Table 9).

**Table 9:** Factors affecting effectiveness of mentee.

The following factors affected my effectiveness as a mentee:	Yes		No	
	n	n/%	n	n/%
Lack of interest in the subject researched	1	6%	17	94%
My struggles with professional identity development	9	50%	9	50%
My psychological and emotional state	9	50%	9	50%
Lack of self-confidence	12	67%	6	33%
Difficulty adapting to changes in the project	11	61%	7	39%
Competing commitments	16	89%	2	11%

*vi*. *Transitioning to a near-peer mentor role (n = 3)*. As mentees progressed, their mentors evaluated the development of their mentees and their suitability as near-peer mentors.

“Yes, I slowly changed from the role of a mentee to near-peer mentor under their guidance. My (senior) mentor's role stayed the same, but with the change in my role as near-peer mentor, my (near-peer) mentor's role also changed to become senior near-peer mentor, whereby my (near-peer) mentor will mentor me to become a better near-peer mentor.”

(Mentee 15)

One of the motivations to take up the role of a near-peer mentor is to pay it forward.

“I became a near-peer mentor for other juniors too- paying it forward”

(Mentee 3)

As the mentees reflected upon their mentoring experiences and growth, the majority of the mentees had a positive mentoring experience, with 94% (n = 17) of them feeling a sense of belonging, teamwork and collegiality.

#### 2) Electronic-mentoring’s role (n = 11)

The primary role of electronic mentoring was to supplement face-to-face meetings (n = 10). Mentees felt face-to-face helped build connections and appreciated the nonverbal communication it provided. As a result, mentees believed electronic mentoring could not replace face-to-face meetings (n = 10).

“Convenient but somewhat more impersonal; meant to complement other aspects of mentoring”

(Mentee 9)

Electronic-mentoring however did enhance access to support and feedback (n = 11).

“Faster/ more efficient communication and dissemination of information”

(Mentee 9)

The asynchronous nature of Electronic-mentoring also facilitated reflective practice (n = 1).

“We do not necessary have to reply immediately and can always take time to reflect prior to replying”

(Mentee 15)

Electronic-mentoring was also seen as an important platform for tracking and disseminating information (n = 1).

“Have details always accessible for better follow up and work.”

(Mentee 4)

#### 3) Near-peer mentor’s role

89% (n = 16) of the mentees felt that the near-peer mentor played a pivotal role in the development of personalized and nurturing mentoring relationship. Effective near-peer mentors were committed, empathetic, non-judgemental, supportive, caring approachable and accessible (n = 17).

“A more junior mentor may be more relatable/approachable to a medical student”

(Mentee 3)

“Perceived as less authoritative/threatening, more collegial environment as responses are more polite, especially helpful climate for novices who may be more shy.”

(Mentee 4)

Near-peers facilitated the timely and personalized support (n = 12).

“One can rely on the secondary mentor should the (senior) mentor be unavailable … multiple mentors may provide a more holistic experience to mentees.”

(Mentee 13)

Near peer mentors provided an alternate source of support (n = 9).

“Spent more time understanding mentee's concerns and sharing valuable experiences and insight”

(Mentee 3)

The near-peer mentors also provided oversight (n = 3).

“(Having a near-peer mentor) definitely helps to keep track of work more closely”

(Mentee 16)

### B. Benefits of CNEP mentoring

Mentees listed the benefits of the program in Table 10.

**Table 10:** Benefits of mentoring program.

I have benefitted from the mentoring program in the following ways:	Yes		No	
	n	n/%	n	n/%
Developed positive values, attitudes and behaviours	15	83%	3	17%
Greater self-confidence	11	61%	7	39%
Improved self-awareness	13	72%	5	28%
Improvement to my psycho-emotional well-being	6	33%	12	67%
I am inspired to pay it forward and mentor.	13	72%	5	28%
Progress in career/ professional development	13	72%	5	28%
Job satisfaction	4	22%	14	78%
Networking	14	78%	4	22%
Increased achievements	13	72%	5	28%

### C. Challenges of CNEP mentoring

There are a number of specific challenges to use of the PMI’s CNEP mentoring approach. These are:

The presence of multiple avenues of communication may become a source of misunderstanding (n = 1).

“Communication and clarification regarding more complex/confusing matters is easier when done face-to-face compared to electronic-mentoring (only)”

(Mentee 13)

Near peers may also be the source of misunderstandings (n = 1).

“(near-peer) and (senior) mentors may have differing views/instructions/perspectives which may confuse medical students”

(Mentee 13)

Use of CNEP mentoring blurred professional and personal boundaries (n = 1).

“Invasion into private time/after hours, but this is universal of communication technologies”

(Mentee 4)

This contributed to stress amongst 67% (n = 12) of mentees and a negative impact upon their work life balance amongst 28% of mentees (n = 5, Table 11).

**Table 11:** Challenges faced as part of mentoring program.

I faced the following challenges in the context of the mentoring program:	Yes		No	
	n	n/%	n	n/%
Disillusionment	1	6%	17	94%
Negative impact on my school/work performance	3	17%	15	83%
Stress	12	67%	6	33%
Negative impact on self-esteem	2	11%	16	89%
Negative impact on work-life balance	5	28%	13	72%
Being treated unfairly	2	11%	16	89%

An expansive program also raises concerns about ethical practice in the mentoring program (Table 12).

**Table 12:** Mentoring ethics.

Mentoring Ethics	Never		Occasionally		Sometimes	
	n	n/%	n	n/%	n	n/%
How often do you face ethical concerns during the mentoring process?	9	50%	7	39%	2	11%
How often do you feel that you have been treated unfairly?	14	78%	3	17%	1	6%
How often do you feel that the mentoring has not taken your interests into consideration?	13	72%	3	17%	2	11%
How often do you feel that the mentoring relationship is focused on serving the mentor's own interests rather than on your development?	13	72%	4	22%	1	6%
How often do you feel that other mentees' were being subjected to mentoring malpractice?	16	89%	2	11%	0	0%

Details of issues faced such as ethical issues, unfair treatment and mentoring malpractice were not expanded upon.

### Phase 3

Based upon advice from the expert team and stakeholders as part of the sixth phase of Braun and Clarke [87]’s approach, comparisons of mentee’s experiences in the CNEP program and accounts of mentoring experiences PMI mentoring set out in Krishna *et al*. [21]’s study were carried out.

Whilst rather superficial given that these comparisons involve qualitative data it is nonetheless reassuring that the themes and accounts detailed in both studies were similar. In addition it lends weight to the validity of the tool designed here given that Krishna *et al*. [21]’s study employed semi-structured interviews that are seen as the ‘gold standard’ for assessing mentoring experiences.

In Krishna *et al*. [21]’s study, the novice mentoring process was seen to evolve in competency-based stages that were described as ‘circumscribed sequential projects’ with ‘specific goals and competency requirements’ that build on one another to achieve an overarching goal. These competency-based stages pivoted on effective communications between mentees and mentors, the establishment of clear expectations, codes of conduct and timelines and the alignment of mentoring goals. The stages of the PMI novice mentoring process were based upon the research process and developed along the course of the research process. These stages include goals setting, data gathering, manuscript writing, submission and responding to the journal’s queries. With PMI CNEP mentoring similarly focused, it is anticipated that these stages are expected to be similar and variations in practice ought to reflect the impact of NP and e-mentoring. Closer scrutiny of these findings will follow.

## Discussion

To address its primary research question, this study has adopted a three phased research approach to circumnavigate the lack of effective assessments tools to evaluate CNEP mentoring. Phase 1 identified and extracted specific activities deemed essential in mentorship programs to evaluate the effects of NP and e-mentoring. Through the Delphi process, a new tool was created and piloted through use of Cognitive Interviews.

Phase 2 saw application of the new tool to capture a mentee’s perspective of the PMI’s CNEP mentoring program. This data is consistent with prevailing accounts of mentoring experiences in novice mentoring programs in Palliative Medicine [1, 4, 10, 12, 13, 20, 21].

In Phase 3 data from the new tool on mentee’s experiences in CNEP experiences were compared with mentee accounts of their experiences in the PMI’s novice mentoring program. These comparisons revealed that CNEP mentoring like the PMI novice mentoring process progressed through the mentoring stages of recruitment, alignment of the mentoring process, data collection, data analysis and preparation for publication. Here similarities between PMI’s previous and present mentoring approaches reaffirm the validity of comparing the findings of the two studies to help discern the impact of changes to the novice mentoring program.

The presence of mentoring stages suggests the presence of two aspects of CNEP mentoring. First is that progress from one stage to another is competency-based and second, CNEP mentoring is dependent upon a structured mentoring approach. In turn these findings have two important implications. A competency-based approach ensures that mentees only progress upon attainment of required skills, reiterating the notion of each stage is a ‘circumscribed sequential projects’ with ‘specific goals and competency requirements’. It also ensures consistent mentoring experiences and clear end points that help alignment of expectations, goals, timelines, expectations and roles and responsibilities [96]. Consistency in the mentoring approach is enhanced by the presence of codes of conduct and the stages of mentoring. The importance of consistency within the mentoring process is underlined by ‘confusion’ amongst mentees when senior and near-peer mentors are not in sync with their advice.

However, it is also clear that the framework can ill afford to be rigid given the need to contend with personalization of the mentoring process. Efforts to personalise the mentoring process was evident in catering meeting and training schedules to the mentee’s abilities, availability and goals. Personalization of the mentoring process was also evident in determining the timelines, codes of conduct, roles and responsibilities of each stakeholder. Ensuring effective balance between a consistent approach and flexibility are personalised, appropriate, specific, timely, longitudinal, accessible and holistic assessments of the mentoring process that inform the administrators and mentors of the adaptations needed.

However, the CNEP mentoring program also throws up new considerations. Unlike the PMI’s novice mentoring program that employed mentee-initiated matching that saw mentee’s select their own mentors, CNEP mentoring employed a combination of mentee-initiated mentor matching and matching of near-peer mentors and mentees. Selection of the near-peer mentor was determined by the senior mentor and was often determined by the research topic of research. There is little evidence of any ill effects to the overall success of mentoring relationships. This may be a reflection of the undiminished primacy of the senior mentor’s role with near-peer mentors providing only practical and psychosocial support, that all near-peer mentors are experienced clinically and from a research perspective, trained and employ the same mentoring approach meaning that there is consistency within the overall mentoring process, the belief that all mentoring interactions are carried out under the oversight of the mentor by virtue of the e-mentoring platform or the fact that e-mentoring allows immediate and easy means of contact with the senior mentor. Many of these possible explanations also raise questions as to whether having NP mentoring and e-mentoring addresses the shortage of mentoring support or merely adds to the senior mentor’s workload. Yet with no failed relationships reported, it would seem that CNEP mentoring appears to be as effective as the PMI’s novice mentoring program.

Once more communications play a critical role in the mentoring process, helping to align expectations, provide feedback and resolve issues. Here it would seem having both the presence of near-peer mentors and e-mentoring support enhanced communication and cultivated a more nurturing mentoring environment and relationship that enhanced the mentoring experience. The combination of NP and e-mentoring and novice mentoring is also seen as a complementary process particularly with data suggesting that neither NP mentoring or e-mentoring was seen as ‘stand-alone’ mentoring approaches. E-mentoring was ‘found to be ‘impersonal’ (Mentee 9) and did not necessarily result in a timely response (Mentee 15) whilst NP mentoring sometimes led to misunderstandings (Mentee 13) and sometimes invasions of private time (Mentee 4). It would seem novice mentoring and oversight and support by the senior mentor was crucial to the overall mentoring process.

However not all the shortfalls facing NP and e-mentoring can be addressed by supplementing them with novice mentoring. The data highlights a number of ethical issues. Perhaps none are so concerning as the worries that 11% of mentees believed that there were ethical issues concerning CNEP mentoring including mentees believing that their near-peer and or senior mentor was motivated by self-interests and/or treated them unfairly. This raises questions as to why 10 of the invited mentees did not participate in the survey with 67% (n = 12) of mentees reported being stressed, 28% (n = 5) experiencing negative effects upon their work life balance and 17% (n = 3) reporting negative impacts upon their clinical work and studies. This is a concern given that CNEP mentoring is lauded for increasing personalised, appropriate, specific, timely, holistic, accessible and longitudinal support and feedback. These findings underline the need for close monitoring of the CNEP program.

The need for effective assessment and oversight of the mentoring process underlines the key role of the host organization. The host organization plays a number of critical roles including recruiting and training mentees and mentors, overseeing and supporting the matching process and structuring the trajectory of the mentoring process around the research process. The other critical role of the host organization lies in its establishment of clear codes of conduct, setting out the roles and responsibilities of mentees, near peer mentors and senior mentors and ensuring effective oversight of the mentoring process. The host organization also play a critical role in supporting mentees particular when some have reported a loss of self-esteem, being treated unfairly and disillusionment. The role, ability and support for the host organization must also be evaluated and must be part of the audit and policing of mentoring processes.

The absence of such holistic assessments re-emphasise the need to move away from reliance upon post-mentoring surveys and snap-shot interviews. It is clear that views and concerns of senior and near-peer mentors and representatives of the host organization are required given that data on their interactions will change the complexion of the discussion and better inform practice and policing of the mentoring program. To enable such a process a mentoring portfolio replete with both mentoring diaries that would capture all interactions between mentee and the mentors and regular input from the mentors on the mentee’s progress and general direction of the progress may be required. For the host organization, feedback loops must be closed and independent assessments of the mentee, senior and near-peer mentors are required. This can capture concerns on the part of any stakeholder throughout the course of the mentoring process and ensure personalised, appropriate, specific, timely, holistic, accessible and longitudinal support and prompt and appropriate training be provided to each party. Evaluations of the host organization itself and the mentoring program as a whole should be carried out regularly to help understand some of the influences upon individual relationships and provide a more complete picture of the mentoring relationship.

Overall, CNEP mentoring does fill many of the gaps in novice mentoring but this approach can only do so when clear lines of communication, effective codes of practice and appropriate oversight is provided. As a result, like novice mentoring, CNEP mentoring needs careful and oversight by the host organization.

## Limitations

Whilst a mixed-method questionnaire approach to appraising mentoring relationships and programs is less resource intensive and offers deeper insights into the CNEP mentoring experiences, it has not yet been validated. Similarly, whilst the themes are similar to those identified in semi-structured interviews of the PMI program, a formal validation program for the new is being planned.

The use of retrospective data at a single time point a variable distance from the end of the mentoring process invites recall bias [97]. The presence of single data points also limits the depth to which it is possible to delve into the various aspects of the mentoring process and the mentoring experiences.

The small sample size and a uniquely structured approach around a research process may limit the applicability of these findings in other settings.

## Conclusion

It is apparent that CNEP mentoring offers a new dimension to mentoring but to realise its full potential CNEP mentoring requires clear mentoring guidelines, mentor, near peer mentor and mentee training, effective holistic and longitudinal assessment of mentoring processes and support [25, 26, 28–32, 35–40, 67, 98–111]. Addressing these issues must be a priority for CNEP program designers and administrators. Fulfilling CNEP mentoring potential must be informed by closer scrutiny of the accounts of ethical issues in mentoring highlighted in recent reviews [22] and supplemented by new systematic reviews of training and coordination of mentoring efforts and mentoring dynamics between mentors and near peer mentors. It is only with these insights and effective support from host organizations can CNEP mentoring play its role in medical education.
